# Medical Inspection of School Children

**Published:** 1908-12-26

**Authors:** 


### MEDICAL INSPECTION OF SCHOOL CHILDREN.

The opening meeting in connection with the last course of lectures and demonstrations on the medical inspection of school children, to be given by Dr. James Kerr, the Chief Medical Officer of the Education Department of the London County Council, will take place at the Rooms of the Society of Medical Officers of Health, No. 1 Upper Montague Street, Russell Square, London, W.C., on January 11. Dr. George Newman, the Chief Medical Officer of the Board of Education, will take the chair and deliver an address prior to the first lecture by Dr. Kerr. The course, which extends over four days, includes the following subjects: "The General Principles of Medical Inspection and Annual Reports," by Dr. Jas. Kerr; "The History and Practice of Medical Inspection," by Dr. C. J. Thomas, of the L.C.C. Education Department; "The Systematic Examination of the Eyes of School Children," by Mr. N. Bishop Harman, illustrated with lantern slides; "Anthropometry," by Dr. F. C. Shrubsall, Secretary of the Anthropometrical Section of the British Association; "Ears," by Mr. P. Macleod Yearsley, of the L.C.C. Education Department; "Teeth" (illustrated with lantern slides), by Mr. Edward Wallis; "Treatment," by Dr. A. H. Hogarth; "The Co-ordination of Medical Inspection with Public Health Work and Annual Reports," by Dr. H. Meredith Richards. Bacteriological demonstrations and demonstrations of special cases will be given by Dr. Jas. Kerr at the Education Offices of the London County Council. Visits will also be made to the Special Schools of the L.C.C., where demonstrations will also be given, and there will be a special lecture on office routine by H. Greer, Esq. An exhibition of special appliances and materials relating to the medical inspection of school children is also being organised. Full particulars as to admission and the times of the lectures may be obtained from Mr. Wm. A. Lawton, Secretary of the Society of Medical Officers of Health, No. 1 Upper Montague Street, Russell Square, London, W.C.

A Photographer's Diary.—We have received a copy of Wellcome's "Photographic Exposure Record and Diary for 1909." This pocket-book contains, besides the necessary ruled pages on which to enter all particulars of every exposure, a mass of information on all branches of photography. The diary can be recommended to those of our profession who are interested in amateur photography.

The directors of the Royal Hospital for Sick Children, Glasgow, have appointed Mr. John James Burnet, A.R.S.A., Glasgow, to be architect; Mr. Archibald B. Watson, I.M., Glasgow, to be measurer; and Dr. Donald J. Mackintosh, M.V.O., of the Western Infirmary, to be their expert adviser and consultant in connection with the erection of the new Children's Hospital on Yorkhill. The plans of a pavilion hospital on the most modern and approved lines, and with the simplest architectural features, are in active preparation, and the work of construction will be commenced as soon as possible.

